# Bioimpedance spectroscopy of breast phantoms

**DOI:** 10.2478/joeb-2025-0007

**Published:** 2025-04-18

**Authors:** Andrey Kuzmin, Viktor Baranov

**Affiliations:** Information and Computing Systems, Penza State University, Penza, Russia; Information and Measurement Systems, Metrology, Penza State University, Penza, Russia

**Keywords:** Electrical bioimpedance, breast cancer, dielectric impedance spectroscopy, breast phantom, Havriliak-Negami model

## Abstract

This article is devoted to the study of the possibility of detecting breast tumors, which can be used for early diagnostics of breast cancer, by the dielectric impedance spectroscopy method. Dielectric impedance spectroscopy is a version of the bioimpedance spectroscopy method, based on the concept of breast tissue as a heterogeneous dielectric, the electrical and mathematical models of which are based on the Havriliak-Negami model. This opens the possibility of getting rid of direct mechanical and electrical contacts between the electrical impedance sensors of the mammograph and the skin. To determine the sensitivity of the dielectric impedance spectroscopy (DIS) method, a series of experiments was performed with phantoms with and without inclusion based on physiological solution (isotonic saline solution) and agar-agar. Using a special capacitive sensor and an LCR meter, the frequency response of the phantoms was determined. Analysis of the statistical characteristics of the differences in the frequency response curves showed significant differences (up to 8% for active R and 4% for reactive X components). The results confirmed the operability of the dielectric impedance spectroscopy method. An important direction for further research is to determine the most informative frequency range.

## Introduction

The problem of early diagnostics of breast tumors is relevant. A serious limiting factor for the effective use of traditional diagnostic methods, such as mammography, is radiation exposure. The attention of many researchers is focused on developing new methods for relatively simple and safe examination of the mammary glands as part of screening examinations [1].

Among such methods, electrical bioimpedance spectroscopy (BIS) is considered the safest and most effective [2]. The sensitivity of BIS is based on significant differences in bioimpedance between normal tissue and tumor [3,4,5].

There are systems based on the principles of bioimpedance tomography that allow obtaining images of internal organs. [6,7,8]. Specialized devices for bioimpedance tomography of the mammary glands are also used, for example, “MEM” [9] and “MEIK” [10]. These devices measure bioimpedance at one fixed frequency of 50 kHz (MEIK) or at two fixed frequencies of 10 kHz and 50 kHz (MEM). The use of such devices requires the participation of a doctor to interpret the obtained images. A modern trend is the use of machine learning in bioimpedance analysis [11,12] and, in particular, to solve the problem of analyzing the results of a tomographic examination without the participation of a doctor [13]. To implement this approach, it is necessary to form a sample containing a significant number of measurement results with different variants of the location and size of the neoplasm inside the object under study.

A promising way to develop a device for examining the mammary gland, which allows eliminating mechanical and electrical contact between the sensor and the human body, is dielectric impedance spectroscopy (DIS). DIS is based on the concept of biological tissue as a dielectric.

The problem of detecting breast tumors based on the concept of measurement object as a dielectric, described by the Cole-Cole equation, is solved in [14]. A tumor in the mammary gland can be detected based on the differences in the complex electrical conductivity of normal and pathological breast tissue [15]. At the initial stage of research on detecting breast tumors, a simulating phantom made of agar, close in complex electrical conductivity, is used [3]. In the study [16] the object of study is described by the Cole-Cole dielectric model.

In a previous study by the authors [17] a concept was formulated for studying the mammary gland with the aim of detecting and localizing neoplasms using various versions of a capacitive sensor, the capacitor of which is filled with a non-uniform dielectric. In this case, it is proposed to describe the object using the Havriliak-Negami mathematical model [18], which, unlike the Cole-Cole model, is proposed for inhomogeneous dielectrics.

To determine the sensitivity of the DIS method to neoplasms in the mammary gland, experimental studies were conducted using phantoms. Phantoms are widely used at the initial stages of research, they can replace biological objects, including in bioimpedance studies [19].

## Materials and methods

To perform experimental studies, phantoms were made based on a water-salt solution and agar-agar. This type of phantom is widely used to study the electrical properties of the mammary gland [3,4]. The concentrations of the components were chosen similar to those used in the studies [3,4]. Two types of phantoms were used in the study:

- a homogeneous phantom (or empty, without heterogeneity);
- a phantom with heterogeneity (including a relatively small object placed inside the phantom with different dielectric properties in comparison with the homogeneous phantom material).

**Fig.1:**
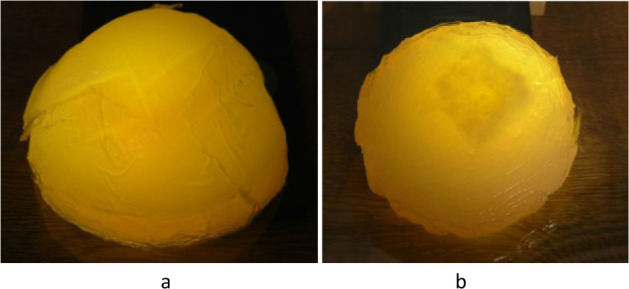
Phantoms with backlight (the light passing through the object): (a) homogenous and (b) with internal heterogeneity.

The material of the internal heterogeneity simulating the neoplasm is similar to the material of the phantom, but differs in the concentration of components, as in the study [4]. The phantoms were given a dome shape, as shown in Fig. 1. The dimensions of the phantoms were chosen to be close to the dimensions of the mammary gland (base diameter about 12 cm, height about 6 cm). The heterogeneity has the shape of a cube with a side length of about 3 cm and is visible in the heterogeneous phantom with backlight in Fig. 1.

When using such phantoms in research, it is necessary to consider that after production the phantoms begin to dry out. After 1–2 days the dimensions and bioimpedance of such phantoms change, and then they cannot be used for experiments.

**Fig.2:**
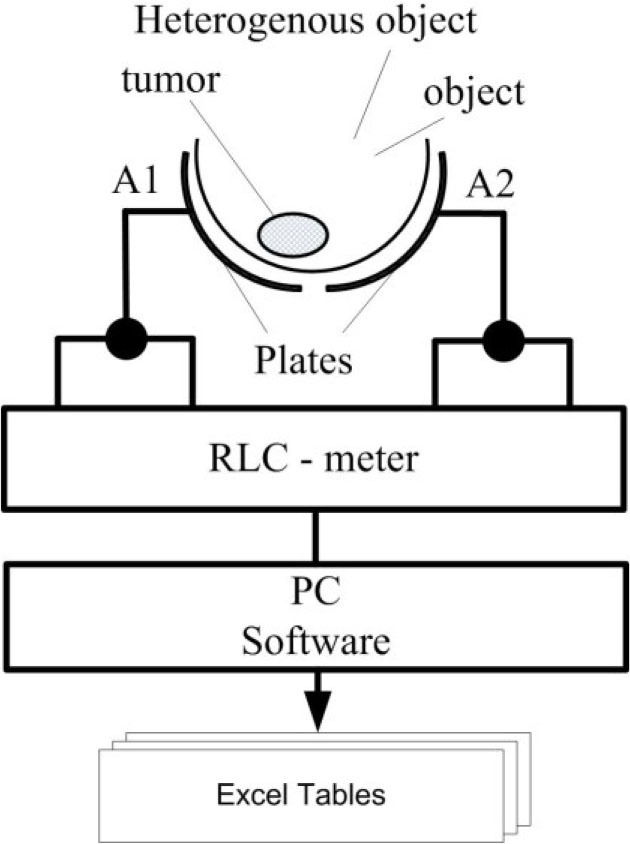
Experimental setup scheme.

For the experimental study of phantoms, a hardware-software complex was assembled, the scheme of which is shown in Figure 2.

The complex consists of a capacitive sensor mounted on an electrostatic mat, a precision LCR meter Keysight Technologies E4980A (https://www.keysight.com), a personal computer and software.

**Fig.3:**
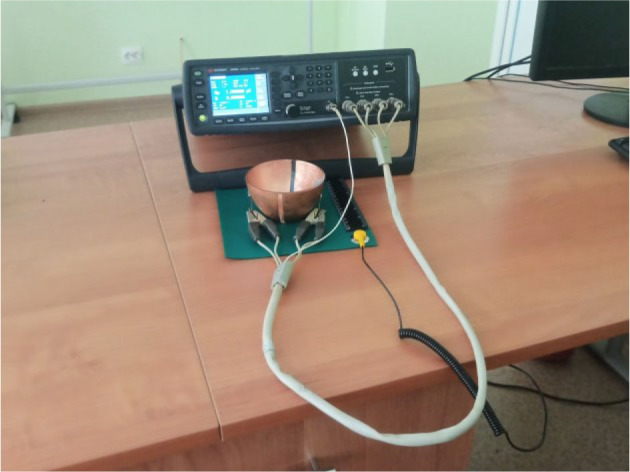
Experimental setup.

A photo of the complex is shown in Fig. 3. The capacitive sensor is a glass bowl, the size and shape of which correspond to the size and shape of the phantom. Two sheets of copper foil are glued to the outer surface of the bowl with a gap, which are the plates of an electric capacitor. A lead is soldered to each sheet, to which the RLC meter is connected.

The software for the complex consists of a specialized program E4980A Data Transfer Program and MS Excel.

The measured parameters of bioimpedance were the active R and reactive X components of the impedance. The measurements were carried out in the List Sweep mode (automatic measurements with a sequential increase in frequency in 10 kHz increments) at 200 frequencies uniformly spaced in the range of 20 Hz – 2 MHz. For each phantom, three series of measurements were carried out to minimize random measurement errors. The data obtained by measurements were processed as follows:

1. The average of three measurement results for each component of bioimpedance at each frequency of the test voltage is calculated.
2. The decimal logarithm is calculated for each of the 200 frequencies of the test signal.
3. Graphs of the amplitude-frequency characteristics of the average components of the bioimpedance of the phantom with simulated tumor and the phantom without it are plotted based on the measurement results at 200 frequencies.
4. The differences between the average components of the bioimpedance of the phantom with and without simulated tumor are calculated at each of the 200 frequencies.
5. The statistical indicators of the difference in the averages for each component of the bioimpedance of the phantom with and without simulated tumor are calculated at each of the 200 frequencies.

The following are used as statistical indicators to evaluate the difference in the bioimpedances of the phantoms:

- Maximum Mean Difference (MMD);
- Mean Square Error (MSE) — the difference between the results of measurements of the bioimpedance component of the phantoms, averaged over the number of frequencies at which the measurement was made, squared.
- Root Mean Square Error (RMSE) — the square root of the mean square difference;
- Mean absolute difference (MAE - Mean Absolute Error) - the sum of all absolute values of the differences in the mean of the phantom measurement results, divided by the number of frequencies at which the impedance components were measured;
- Mean absolute error in percent (MAEP - Mean Absolute Error Percent) - the sum of all absolute values of the differences in the mean of the phantom impedance component measurement results, divided by the sample size (the number of frequencies at which the impedance component was measured), expressed as a percentage of the mean of the impedance component measurement results.
- Square difference of the mean of the phantom impedance component measurement results at each frequency.

### Ethical approval

The conducted research is not related to either human or animal use.

## Results

The results of measurements of the bioimpedance components of phantoms at selected frequencies of 200 values: 20 Hz, 100 Hz, 1 kHz, 10 kHz, 50 kHz, 100 kHz, 500 kHz, 1 MHz, 2 MHz are presented in Table 1. Negative values of the X component indicate a capacitive characteristic of this bioimpedance component.

**Table 1.** Experimental results.

**Frequency**	**20 Hz**	**10 kHz**	**50 kHz**	**100 kHz**	**200 kHz**	**500 kHz**	**1 MHz**	**1.5 MHz**	**20 MHz**
**Homogenous phantom, GΩ**									
R	7.982	4.844	3.865	3.439	3.040	2.548	2.198	1.986	1.831
X	−8.091	−5.877	−5.207	−4.912	−4.616	−4.224	−3.927	−3.755	−3.634
**Heterogenous phantom, GΩ**									
R	8.060	4.843	4.049	3.657	3.242	2.682	2.275	2.059	1.887
X	−7.768	−5.917	−5.257	−4.967	−4.673	−4.281	−3.984	−3.810	−3.689
**Differences, GΩ**									
R	0.078	0.001	0.185	0.218	0.202	0.138	0.077	0.073	0.055
X	0.322	0.040	0.049	0.055	0.057	0.058	0.057	0.055	0.055

**Fig.4:**
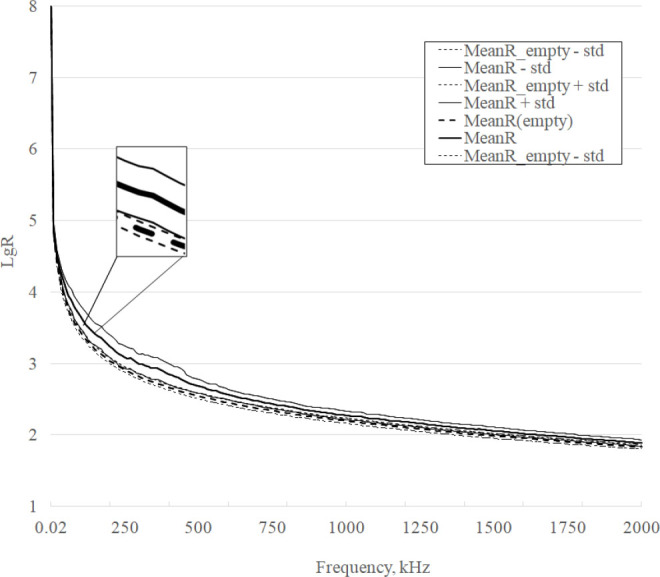
R curves for homogenous and heterogeneous phantoms.

The frequency response curves in the range of 20 Hz – 2 MHz of the active dielectric component parameter R of the phantoms are shown in Fig. 4. Each R curve represents logarithmic data averaged over 3 measurement series for homogeneous and inhomogeneous phantoms, which allows us to reduce the random measurement error.

It is evident from Figure 4 that approximately at the point corresponding to a frequency of 40 kHz the curves begin to separate significantly and this difference increases with increasing frequency, and approximately at the point corresponding to a frequency of 800 kHz this difference begins to decrease monotonically, although they do not intersect further until the highest frequency of the range is reached.

**Fig.5:**
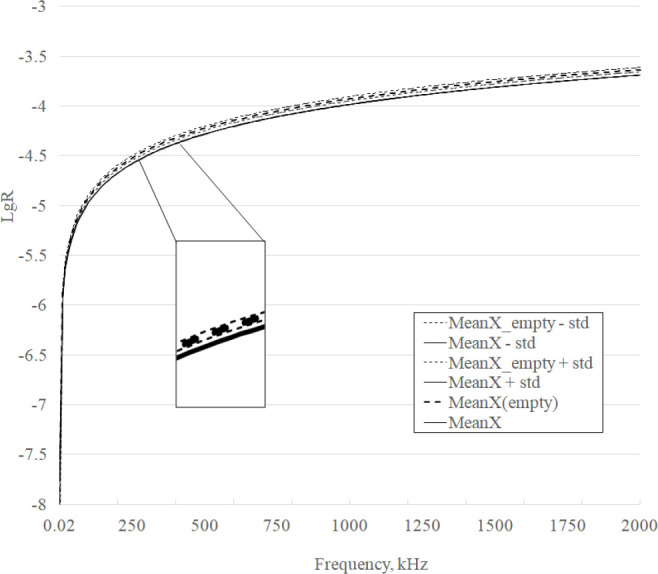
X curves for homogenous and heterogeneous phantoms.

The frequency response curves in the range of 20 Hz – 2 MHz of the parameter of the reactive dielectric component X of the phantoms are shown in Figure 5. Similar to Figure 4, each curve X represents logarithmic data averaged over 3 measurement series for homogeneous and inhomogeneous phantoms, which allows reducing the random measurement error.

It is evident from Figure 5 that approximately at the point corresponding to a frequency of 60 kHz the curves begin to separate significantly and this difference increases with increasing frequency, and approximately at the point corresponding to a frequency of 700 kHz this difference begins to decrease monotonically, although they do not intersect further until the highest frequency of the range is reached.

**Fig.6:**
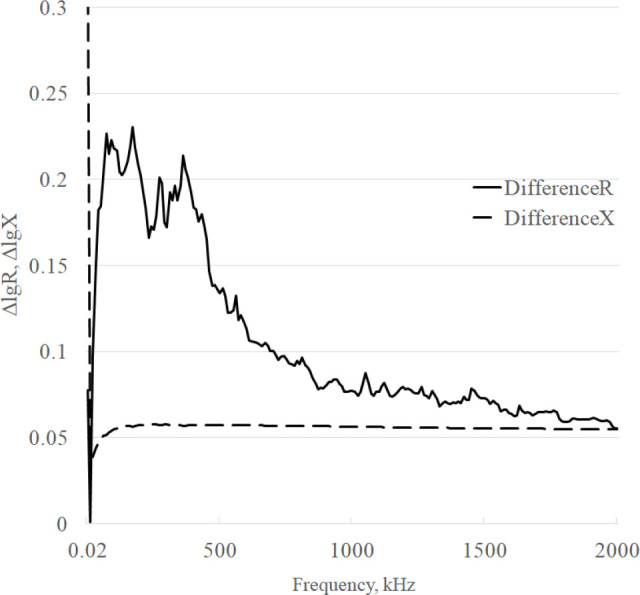
Difference curves.

The curves of the differences in the frequency response of the averaged components R and X in the range of 20 Hz – 2 MHz are shown in Fig. 6.

The “jaggedness” of the graphs in Fig. 6, taking into account the relatively small differences, indicates that there is some random error in the measurements. Visual analysis of the difference graphs shows that the maximum differences are observed for the R component up to the frequency of approximately 500 kHz.

The values of the statistical indicators of the difference between the phantoms are given in Table 2.

**Table 2.** Statistic parameters.

Parameter	R	X
Max. Mean difference	7.86%	3.98%
MSE	0.010	0.004
RMSE	0.12	0.06
MAE	0.11	0.06
MAEP	0.04	0.04
Square difference	21.04	11.49

The data presented in Table 2 show that for all statistical parameters studied, the values for the active component are approximately twice as large as for the reactive component (except for MAEP). This indicates a lower sensitivity of the reactive component to the presence of inclusions. The information content of statistical parameters in terms of distinguishing the frequency response curves of the phantoms studied is comparable.

## Discussion

The general appearance of the obtained frequency response (see Figs. 4, 5) corresponds to the appearance of the frequency response of normal (control) breast tissue and tumor, Fig. 3 and Fig. 4 [1], which, according to the authors, confirms the correctness of the experimental results. There are large values on the DifferenceR graph (see Fig. 6) of the difference frequency response of the active component of bioimpedance in the low-frequency region. The authors explain it by the high modulus of bioimpedance (> 5 GΩ). At the test voltage of 2 V, the measuring current decreases to units of pA and becomes comparable with the level of intrinsic noise and the quantization error of the device. With an increase in the frequency of the test signal measurement, the amplitude of the peaks decreases. At frequencies above 10 kHz, where the modulus of bioimpedance decreases significantly the amplitude of the peaks becomes low.

A significant difference in the mean values of the active and reactive components of the bioimpedance of phantoms is demonstrated in Figs. 4 and 5. The calculated p-values in accordance with the Student’s criterion confirm the significance of the differences in the experimental frequency response curves: for the R and X components p < 0.002. Taking into account the values of standard deviations, the parameters in the range of 40–130 kHz are most reliably distinguished.

The possibility of detecting a tumor by R component measurements in the range of 40–130 kHz is confirmed by partial overlap with the range of 100 kHz – 100 MHz, in which the results were obtained in the work [14], with the range of 0.488–1000 kHz [15] and with the range of 10 kHz to 1 MHz [20]. The informativeness of this frequency range in solving the problem of detecting neoplasms is confirmed by the fact that the study of the mammary gland by the method of bioimpedance tomography is carried out at the frequency of 50 kHz [9,10]. In this case, the maximum difference in the frequency response is observed at a frequency of 360 kHz and is about 8%. This allows us to potentially consider a wider range of 40–630 kHz in further studies.

The difference of frequency response values of the reactive component of the bioimpedance of the phantoms is almost constant in the informative range from 60 kHz up to 2 MHz, the DifferenceX graph has no outliers, but the sensitivity to the presence of inclusion is two times lower, 4% in comparison with the active component.

The analysis of the calculated statistical indicators describing the difference in the frequency response of the active and reactive components of the bioimpedance of homogeneous and inhomogeneous phantoms (Table 2) showed that the highest sensitivity to the appearance of inhomogeneity have the indicators “Maximum difference of the average active component of bioimpedance (R)” - 8% and “Difference of the squares of the average of the results of measurements of the active component of bioimpedance (Square difference R)” - 21.0 GΩ^2^.

The next stage of the research is:

- to set the upper limit of the informative range of the test voltage frequency at 630 kHz for R the component measurement;
- to conduct a study aimed at identifying the causes and developing methods for minimizing abnormal peak values of the differential frequency response of the active component of the bioimpedance in order to expand the informative range to the low-frequency region of less than 1 kHz, where there is high sensitivity to the presence of heterogeneity in the phantom;
- to develop and manufacture versions of a capacitive sensor with 4 plates of different shapes (sector, horizontal strip) and conduct experimental studies of localization and determination of the size of heterogeneity in a heterogeneous phantom.

The data in Table 2 indicate that the values of the active component of the complex resistance are more sensitive to changes. At the same time, the analysis of the curves in Figs. 4 and 5 shows that, taking into account the standard deviations of the parameters, the frequency response curves of the reactive component intersect less. This allows us to conclude that both components of the complex resistance must be taken into account to detect new formations. Determining an integral indicator that takes both components into account is a topic for further research.

## Conclusion

The experimentally revealed differences in the bioimpedances of homogeneous and inhomogeneous phantoms, according to the authors, are quite significant in the frequency range of 40 kHz – 130 kHz for the R component and 60 kHz – 2 MHz for the X component, in particular, the statistical indicator “Maximum difference in the average active component of bioimpedance” was 8%, and the indicator “Difference in the squares of the average results of measurements of the active component of bioimpedance” was 21.0 GΩ^2^. The experimental results allow us to consider the dielectric spectroscopy method as promising for the development of devices for screening examination on its basis. This determines the feasibility of moving on to the next stage of the study: studying the possibility of detecting, localizing and determining the size of breast tumors.
